# Long-term high-fructose high-fat diet feeding elicits insulin resistance, exacerbates dyslipidemia and induces gut microbiota dysbiosis in WHHL rabbits

**DOI:** 10.1371/journal.pone.0264215

**Published:** 2022-02-23

**Authors:** Michelle Moughaizel, Elie Dagher, Amin Jablaoui, Chantal Thorin, Moez Rhimi, Jean-Claude Desfontis, Yassine Mallem

**Affiliations:** 1 Nutrition, PathoPhysiology and Pharmacology (NP3) Unit, Oniris, Nantes Atlantic College of Veterinary Medicine, Food Science and Engineering, Nantes, France; 2 Laboniris, Oniris, Nantes Atlantic College of Veterinary Medicine, Food Science and Engineering, Nantes, France; 3 Institut Micalis, INRAE, AgroParisTech, Université Paris-Saclay, Jouy-en-Josas, France; University of Utah, UNITED STATES

## Abstract

The metabolic syndrome (MetS) has become a global public health burden due to its link to cardiovascular disease and diabetes mellitus. The present study was designed to characterize the metabolic and cardiovascular disturbances, as well as changes in gut microbiota associated with high-fructose high-fat diet (HFFD)-induced MetS in Watanabe heritable hyperlipidemic (WHHL) rabbits. Twenty-one Watanabe rabbits were assigned to a control (n = 9) and HFFD (n = 12) groups, receiving a chow diet and a HFFD, respectively. During a 12-weeks protocol, morphological parameters were monitored; plasma fasting levels of lipids, glucose and insulin were measured and a glucose tolerance test (GTT) was performed. HOMA-IR was calculated. Cardiac function and vascular reactivity were evaluated using the Langendorff isolated heart and isolated carotid arteries methods, respectively. 16S rRNA sequencing of stool samples was used to determine gut microbial composition and abundance. HFFD-fed Watanabe rabbits exhibited increased fasting insulin (*p* < 0.03, 12^th^ week vs. Baseline), HOMA-IR (*p* < 0.03 vs. Control), area under the curve of the GTT (*p* < 0.02 vs. Control), triglycerides (*p* < 0.05, 12^th^ week vs. Baseline), TC (*p* < 0.01 vs. Control), LDL-C (*p* < 0.001 vs. Control). The HFFD group also displayed a significant decrease in intestinal microbial richness, evenness and diversity (*FDR* < 0.001, *FDR* < 0.0001, *FDR* < 0.01, respectively vs. Control group) and an increase in its *Firmicutes*/*Bacteroidetes* ratio (R = 3.39 in control vs. R = 28.24 in the HFFD group) indicating a shift in intestinal microbial composition and diversity. Our results suggest that HFFD induces insulin resistance and gut microbiota dysbiosis and accentuates dyslipidemia; and that, when subjected to HFFD, Watanabe rabbits might become a potential diet-induced MetS animal models with two main features, dyslipidemia and insulin resistance.

## Introduction

The metabolic syndrome (MetS) includes abdominal (central/visceral) adiposity, insulin resistance (IR), impaired glucose tolerance, arterial hypertension, and dyslipidemia [1]. Each component of the MetS is considered as an independent risk factor for cardiovascular disease [2]. When combined these factors contribute to elevated risk of a spectrum of cardiovascular conditions e.g. cardiac and endothelial dysfunction and atherosclerosis [2–4]. The MetS has become the major global health hazard of the modern world due to a worldwide shift towards a western lifestyle [5, 6]. It is estimated that over 1 billion people worldwide are affected by this syndrome [5]. The continuous and rapid rise in the global prevalence of MetS are attributed to increased consumption of food and beverages that are rich in energy, fat, and added sugar, on one hand and to decreased physical activity/sedentary lifestyle, on the other hand [5–8]. It has been demonstrated that chronic consumption of a western diet, rich in sugar and saturated fat, increases the risk of developing IR [9, 10], dyslipidemia and obesity [11, 12], three main components of MetS [13, 14].

The gut microbiota plays an important role in the regulation of host metabolic functions and in the control of energy homeostasis through modulating energy absorption, gut motility, appetite, glucose and lipid metabolism, as well as hepatic fatty storage [15, 16]. However, available evidence from both human and animal models studies also suggest that deleterious changes to the composition of gut microbiota, referred to as gut dysbiosis, exert a significant role in the pathogenesis of the MetS [16–19]. Moreover, it was found that germ-free animals are resistant to high-fat diet-induced obesity and IR indicating that gut microbiota likely participates in the development of the MetS [20]. Nevertheless, the exact key mechanisms underlying the implication of gut microbiota dysbiosis in metabolic and CVD are just yet starting to be delineated and are still to be thoroughly investigated.

The pathogenesis of the MetS is not fully understood due to its complexity and to the interconnection between its components [21]. Working on suitable animal models that mimic human MetS is of utmost importance to gain a deeper insight into the development of this syndrome. Even though numerous animal models of MetS have been established, those combining dyslipidemia and IR without obesity as a main component, are scarce. Accordingly, data on the association between dyslipidemia and IR in the MetS are less available, and whether this association is independent of other components of the MetS, has not been reported. Similarly, to date most existing studies have linked the intestinal dysbiosis to obesity but few have investigated its association with IR and dyslipidemia.

The aims of the present study were 1) to investigate/characterize the changes in metabolic and cardiovascular parameters, encountered during the MetS and mainly related to combined IR-dyslipidemia and 2) to explore gut microbiota compositional changes that are associated with this combination, independently from other components of the MetS, i.e. obesity. We exposed WHHL rabbits to a HFFD during 12-weeks protocol. It is to be noted that in general and unlike rodents, rabbits have a human-like lipoprotein metabolism (higher LDL than HDL) and therefore represent the best candidates in terms of animal models for the study of lipid/lipoprotein metabolism and atherosclerosis [22–24]. The WHHL rabbit, in particular, is an animal model of human familial hypercholesterolemia (FH) due to a genetic defect in its low-density lipoprotein receptor (LDLr); that leads to a delayed clearance of LDL particles from the circulation and consequently to hyperlipidemia and spontaneous atherosclerosis [22, 25]. Furthermore, exposing animals to high fructose and/or high fat diet has revealed to be successful in inducing several components of the MetS [26]. It has been demonstrated that one of the mechanisms behind this success is the expansion of the intestinal villus length/surface area of the gut that eventually leads to increased nutrient absorption and adiposity [27–29].

## Materials and methods

### Animals and diet

Twenty-one Watanabe heritable hyperlipidemic (WHHL) rabbits (male and female) aged 22 to 26 weeks and weighing 2.7–3 kg (at the end of the protocol) were used in all experiments. Rabbits (CEGAV, France) were housed individually in a room maintained at constant temperature of 21°C and humidity (>45%), under a 12h dark/light cycle with free access to water and food. Rabbits were randomly divided into two groups, a control group (n = 9) fed a standard chow diet (3410.PS.S10; SERLAB, France) and a HFFD group (n = 12) fed a chow diet that is supplemented with 30% fructose and 10% coconut oil (91% saturated fatty acids) (SERLAB, France). The protocol lasted for 12 weeks. In order to avoid the excessive weight gain, we used a diet that is rich in sugar and fat but with less protein and fibers, compared to a standard chow [26].

The study was conducted in accordance with the National Institute of Health Guide for the Use and Care of Laboratory Animals guidelines and they are all conducted with the approval of the Ethics Committee of Pays de la Loire (APAFIS n° 10722).

### Morphological parameters

Weight and abdominal circumference were weekly measured to monitor the animal’s weight gain and growth.

### Measurement of plasma lipids, glucose and insulin levels

After an overnight fasting, cannulation of the auricular artery (24 G, Vasofix®) and blood withdrawal were performed under local anesthesia (EMLA cream; 2.5% lidocaine, 2.5% prilocaine) at the beginning and end of the protocol. Samples were centrifuged (10000g, 15 minutes, 4°C) and the plasma was stored at -80°C. We used an enzymatic colorimetric method for the quantitative determination of total cholesterol (TC), triglycerides (TG), free fatty acids (FFA) and high-density lipoprotein cholesterol (HDL-C) plasma levels. The dosages were performed according to the supplier’s instructions (DiaSys enzymatic commercial kits). Meanwhile, low-density lipoprotein (LDL-C) levels were calculated using the Friedewald’s equation, LDL cholesterol (g/L) = Total cholesterol–HDL-cholesterol–(Triglycerides/5) [30]. We used a sensitive rabbit insulin ELISA kit sandwich assay (Crystal Chem High Performance Assays, USA) for the quantitative determination of insulin plasma levels.

### Intravenous glucose tolerance test

To examine the effects of HFFD on glucose metabolism and insulin response, an intravenous glucose tolerance test (IVGTT) was performed, at the beginning and end of the protocol. After an overnight fasting, cannulation of the auricular vein (24 G, Vasofix®) was performed under local anesthesia (EMLA cream; 2.5% lidocaine, 2.5 prilocaine). An intravenous glucose solution (0.6 g/kg body weight) was then injected. Blood samples were taken and glucose was measured at 0, 15, 30, 45, 60, 90 and 120 min, post glucose injection. Blood glucose levels were measured using one drop of whole blood, from the marginal ear vein via a glucose meter (ONETOUCH®VERIO®; © 2012 LifeScan, Inc.). Samples were stored on ice and centrifuged (10000g, 15 minutes, 4°C) to later measure plasma insulin concentrations using rabbit insulin ELISA kit (Crystal Chem High Performance Assays, USA). The area under the curves (AUC_Glu_ and AUC_Ins_) were calculated using GraphPad PRISM® software (version 8.1). Meanwhile, Homeostasis Model Assessment of basal Insulin Resistance (HOMA-IR) was calculated using the following equation: [(fasting plasma glucose (mmol/l) x fasting plasma insulin (μU/ml))/22.5] [31].

### Isolated heart preparation, Langendorff method

After the animal was anesthetized using an intravenous Sodium Pentobarbital injection (40 mg/kg), the thorax was cut and opened. The animal was sacrificed by exsanguinating the abdominal aorta. The heart was rapidly excised and transferred into ice-cold filtered (using a filter funnel) Krebs’ solution, containing (mM): NaCl 118.3, NaHCO3 20.0, KCl 4.7, MgSO47H2O 1.2, KH2PO4 1.2, glucose 11.1, EDTA 0.016 and CaCl2 2.5. Afterwards, the heart was cannulated (4 mm aortic cannula, ADinstruments) and perfused at a constant flow-rate (22–28 mL/min) with a Krebs solution that was constantly bubbled with 95% O2 and 5% CO2 at 37°C. A pressure transducer located above the aorta recorded the perfusion pressure (PP), considered as an index of coronary vasodilation. Then a de-ionized water-filled latex balloon connected to another pressure transducer was inserted through the mitral valve into the left ventricle to record the left ventricular pressure (LVP) as an index of cardiac contractility. The heart was allowed to stabilize for 20 minutes before performing non-cumulative concentration-response curves (non-CCRCs) to isoproterenol (10^−9^ to 10^−6^ M), a nonselective β-adrenergic agonist. Data acquisition and recording was achieved using Powerlab 8/35 and LabChart 7.0 software (ADInstruments). The left ventricular pressure (LVP) and coronary perfusion pressure (PP) were continuously measured during the experiment. The left ventricular developed pressure (LVDevP) was calculated as follows: LV systolic pressure—LV end-diastolic pressure.

### Vascular reactivity measurement

Immediately after the animal was sacrificed by exsanguination, the carotid artery was removed and dissected free of fat and connective tissue and placed in a cold Krebs’ solution (see composition above). The artery was cut into 4-5mm ring segments that were then mounted and suspended in 10 mL organ baths containing Krebs’ solution, upheld at 37°C and constantly bubbled with carbogen (95% O2 and 5% CO2). The isometric tension was continuously measured using an automated isometric transducer system (EMKAbath4, EMKA technologies, France). Data were recorded using an acquisition software (iox version 2.9.5.20). The arterial rings were gradually loaded to an initial 2 g tension and allowed to equilibrate for 60 minutes. The viability of the vessels was evaluated using an 80 mM KCl solution. Then, the presence of intact endothelium was verified by adding acetylcholine (Ach, 10^−6^ M) to phenylephrine (Phe, 3.10^−6^ M)-precontracted rings. The rings with less than 60% relaxation were considered non-reliant and thus, were eliminated. Cumulative concentration-response curves (CCRCs) to Phe, an α_1_-adrenoceptor agonist (10^−9^ to 3.10^−5^ M) were constructed. CCRCs to Ach, a muscarinic receptor agonist (10^−9^ to 3.10^−5^ M) and to insulin (Ins, 10^−9^ to 3.10^−6^ M) were built on Phe-precontracted rings. The contraction and relaxation percentages were calculated in relation to the maximal response from the precontraction produced by KCl- and Phe-precontractions, respectively.

### Histology and Immunohistochemistry

We evaluated the effect of HFFD on the development and evolution of atherosclerosis in Watanabe rabbits. Immediately after animal sacrifice, the area preceding the aortic arch of the cranial thoracic aorta was removed and fixed in 10% neutrally buffered formalin. After fixation, samples were dehydrated, embedded in paraffin and cut into serial 4μm thick sections before they finally underwent staining.

For histological analysis, sections were stained with hematoxylin and eosin safranin (HES) and with Green Masson’s trichrome and Orceine to detect collagen and elastic fibers, respectively. Other sections were immunohistochemically stained with macrophage antibody (clone RAM11, mouse monoclonal, 1:1200 dilution, Dako) and muscle actin antibody (clone HHF35, mouse monoclonal, 1:50 dilution, Dako) to detect macrophages (Mφ) and smooth muscle cells (SMCs), respectively.

To determine the extent of atherosclerotic lesions both the percentage of the lesion area and the degree of intimal thickening were determined using an image analysis system (FIJI, Image J®). The average intima thickening was calculated as follows: the area of intimal lesion was divided by the length of media. Lesions were classified, by a certified veterinary pathologist, into different types (I, II, III, IV, V and VI) according to the guidelines of the American Heart Association [32]. In order to compare the stage of lesions, type II and III plaques were classified as early lesions whereas type IV and V plaques were classified as advanced lesions. The FIJI software was also used to quantify elastin fibers, macrophages and SMCs, by calculating the percentage of immunostaining in lesion area. An algorithm could not be used for the collagen deposition (fibrosis). The latter was graded, by a veterinary pathologist, along with the presence of extracellular lipid, fibrous cap, mineralization and lipid core. The grading was performed using a semi-quantitative scale from 0 to 4 in order to better characterize the different types of plaques as previously described [32].

### Pulse wave velocity measurement

To assess the arterial stiffness, two sets of pulse wave velocity (PWV) measurements were performed, one at the beginning and another, at the 12th week of the protocol. Two piezoelectric sensors (ADInstruments) were placed, one on the auricular artery and another on the tail one, of awake animals. Recordings of pulse waves were made during 10 min with the software (ADInstruments LabChart v8.1.5). The measurement point (the foot of each pulse wave) was determined using the second derivative of the pulse wave signal. Both the distance between the two sensors and that between the auricular artery and the sternal manubrium, were noted each time. Then, the Δx was calculated by substracting the two distances. The time interval (Δt) between the foot of the auricular waveform and that of the tail waveform was measured by the software. The PWV was calculated using the formula PWV (m.s-1) = Δx/Δt [33].

### Gut microbiota analysis

Fecal samples were collected at the beginning and end of the protocol. Upon their collection, samples were immediately immerged in liquid nitrogen and were then stored at -80°C. The genomic DNA (gDNA) was extracted from fecal samples using Guanidium Thiocyanate and mechanic disruption with bead-beating method as previously described by Godon et al. (1997) [34]. The V3-V4 region of the purified DNA was amplified during 30 amplification cycles, at 65°C, using the forward primer F343 (CTTTCCCTACACGACGCTCTTCCGATCTACGGRAGGCAGCAG) and the reverse primer R784 (GGAGTTCAGACGTGTGCTCTTCCGATATTACCACC). The 510 bp amplicons were, then purified and all non-specific primers were removed. A second 12 cycles PCR was performed by adding a home-made 6 bp index to the reverse primer R784 (AATGATACGGCGACCACCGAGATCTACACTCTTTCCCTACACGAC) and by using a modified reverse primer (CAAGCAGAAGACGGCATACGAGAT-index-GTGACTGGAGTTCAGACGTGT) via the IlluminaMiseq® technology. The purification and loading of the resulting products onto the Illumina MiSeq cartridge were performed according to the manufacturer’s instructions. PhiX was used to check the quality of the run.

The sequenced samples were analyzed using the bioinformatics pipeline FROGS (Find Rapidly OTU with Galaxy Solution) [35]. Using Flash we assembled the sequences trimmed for adaptors [36]. PCR primers were removed and sequences with sequencing errors in the primers were eliminated (cutadapt) [37]. For each sample chimera were removed using vsearch and Uchime [38, 39].The total number of reads counted 11 837 508 versus 10 927 648 count of minimum reads, kept after cleaning data. Reads were clustered into Operational Taxonomic Units (OTUs) at the 97% identity level using Swarm with a cut-off value of 0.03 [40, 41]. A reference sequence for each OTU was opted and assigned to different taxonomic levels (from Kingdom to species) using the Silva database [42] and the RDP classifier [43].

To investigate α and β diversities and microbiota composition differences between the two groups, all samples were rarefied to the same depth before analysis with the phyloseq R package with a minimum reads number of 45 554. OTU Richnness (Observed) and diversity (Shannon and Simpson) within samples was used to calculate alpha diversity. Significant differences between studied groups were assessed using Mann Whitney test. Community compositions between samples in the different groups were assessed using permutational multivariate analysis of variances (PERMANOVA). Significance was checked using Adonis statistical tests (vegan package of R) to evaluate the distances at 9999 permutations between the two groups. The abundances of given microbial families were calculated by gathering all OTUs belonging to these families. Difference in microbiota composition at phylum, family and genus level, between the studied groups was assessed using Mann Whitney test. Benjamini‐Hochberg corrections (BH) were used to avoid false positives (significance threshold = 0.05) [44]. An adjusted p value (FDR) of less than 0.05 was considered statistically significant.

### Statistical analyses

Data were expressed as a mean ± SEM. All graphs were performed using PRISM® software (version 8.0.1). We used repeated measures Two-way ANOVA for multiple group comparisons. Linear Mixed effect (LME) model was used to analyze data from non-CCRCs (isolated heart) and CCRCs to insulin. Meanwhile, Non-linear Mixed Effect model (NLME) was used to assess data from CCRCs to Phe and Ach [45]. Contraction and relaxation were expressed as the percentage relaxation of the KCl- and Phe-induced precontraction, respectively. The efficacy (E_max_) and the potency (pD_2_), respectively, representing the maximum effects and the negative logarithm of the concentration producing 50% of the maximum effect were determined for each of the CCRCs.

Mann Whitney statistical test with a *p* value correction (FDR) according to the Benjamini and Hochberg was used to study the composition of the fecal microbiota. Comparative analysis of the microbial composition and abundance were performed at the phylum, family and genus levels.

R software was used to evaluate data from CCRCs, non-CCRCs and from gut microbiota analyses. A *p* value of less than 0.05 was considered statistically significant for all results.

## Results

### Weight gain, lipid profiles and glucose and insulin metabolism

After 12 weeks of HFFD-feeding, the body weight significantly increased in the both the control and HFFD groups when comparing 12^th^ week values to baseline values (*p* < 0.0001 and *p* < 0.01, respectively). Whereas, at the 12^th^ week of the protocol there was not any significant difference in body weight when comparing the two groups together. Similarly, after 12 weeks of the protocol, the increase in weight was significant in both the control and the HFFD groups when comparing 12^th^ week values to baseline values (*p* < 0.0001 and *p* < 0.001, respectively) but not between the two groups (HFFD vs. Control) at the end of the protocol (12th week). Both weight and abdominal circumference parameters significantly (similarly) increased in both groups (12^th^ week vs. Baseline) (Table 1).

**Table 1:** Results of weight, abdominal circumference, glycaemia, insulin and plasma lipids.

Groups			
Measurements	Control (n = 9) HFFD (n = 12)			
	Baseline 12^th^ week		Baseline 12^th^ week	
Weight (Kg)	2.00 ± 0.10	2.97 ± 0.10 ^$ $ $ $^	2.30 ± 0.05	2.70 ± 0.12 ^$ $^
Abdominal circumference (cm)	31.78 ± 1.17	38.78 ± 0.76 ^$ $ $ $^	31.81 ± 0.88	36.45 ± 0.62 ^$ $ $^
Fasting glycaemia (mg/dL)	98.00 ± 1.88	96.00 ± 3.06	101.00 ± 6.60	106.00 ± 7.54
Fasting insulinemia (ng/mL)	0.37 ± 0.08	0.31 ± 0.07	0.30 ± 0.04	1.15 ± 0.45^$^
Triglycerides (g/L)	1.40 ± 0.30	1.75 ± 0.35	1.76 ± 0.30	2.65 ± 0.39 ^$^
TC (g/L)	6.72 ± 0.50	6.53 ± 0.80	7.40 ± 0.50	11.05 ± 0.88 ^$ $^ **
HDL-C (g/L)	0.21 ± 0.03	0.22 ± 0.03	0.19 ± 0.02	0.26 ± 0.03
LDL-C (g/L)	6.23 ± 0.45	5.96 ± 0.70	6.87 ± 0.50	10.25 ± 0.85 ^$ $^ ***
FFA (mmol/L)	0.83 ± 0.20	0.65 ± 0.10	1.02 ± 0.21	0.70 ± 0.11

TC = total cholesterol, HDL-C = high-density lipoprotein, LDL-C = low-density lipoprotein, FFA = free fatty acids. Data are represented as mean ± SEM

$ *p* < 0.05

$ $ *p* < 0.01

$ $ $ <0.001 and

$ $ $ $ *p* < 0.0001 vs. Baseline.

** *p* < 0.01

*** *p* < 0.001 vs. Control.

We additionally examined lipid profile modifications (Table 1). Both TC and LDL-C significantly increased in the HFFD group but remained unchanged in the control group when comparing 12th week values to baseline values (*p* < 0.02). These levels also significantly increased when comparing the two groups together (HFFD vs. Control) at the end of the protocol (*p* < 0.01 for TC and *p* < 0.001 for LDL-C). TG levels significantly increased in the HFFD group, but not in the control group, when comparing 12th week levels to baseline levels (12th week vs. Baseline, *p* < 0.05). Meanwhile, no change was observed in terms of HDL and FFAs neither when comparing the 12th week levels to the baseline levels nor when comparing the two groups to each other at the end of the protocol.

**Fig 1:**
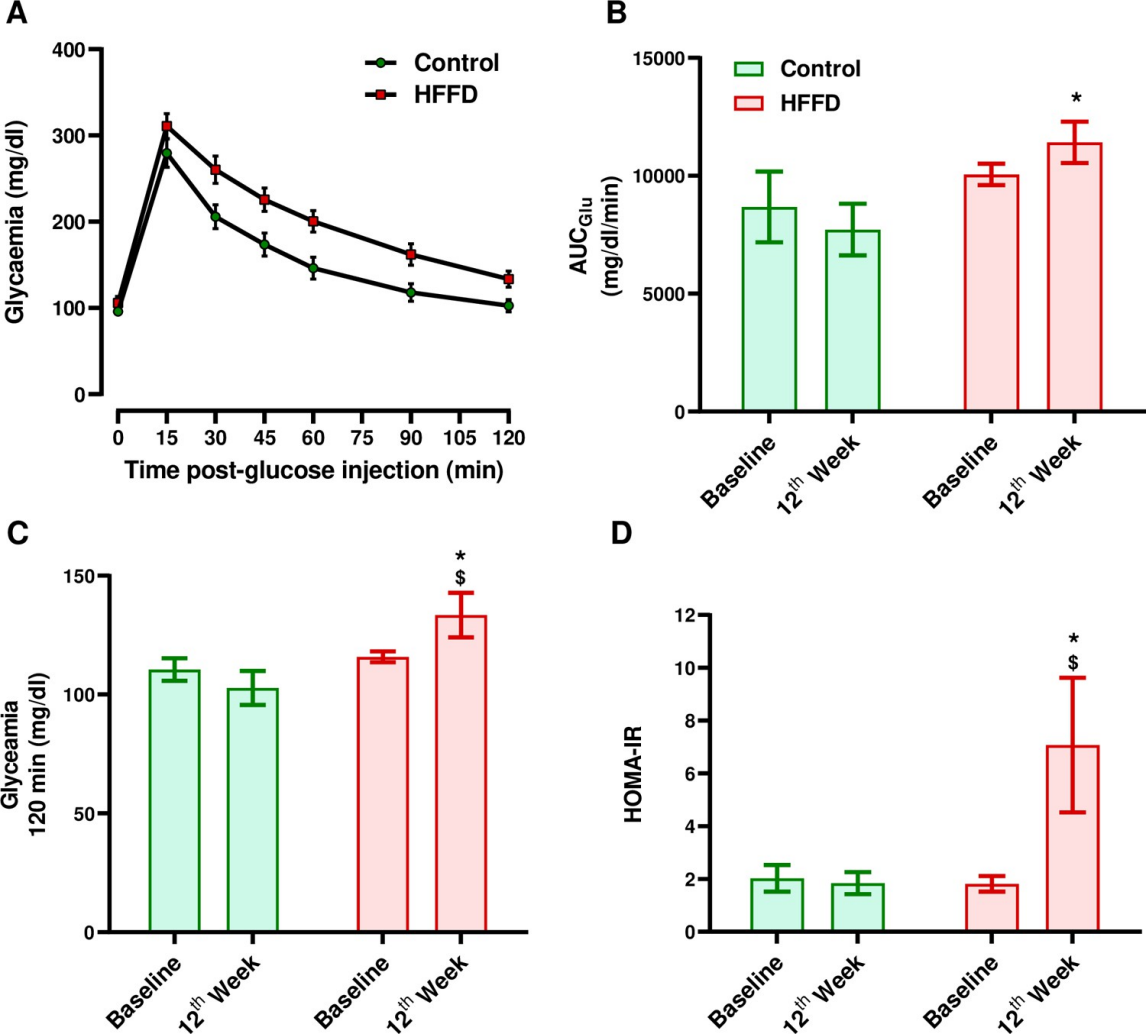
Effect of HFFD feeding on glucose and insulin metabolism. Glucose metabolism was evaluated by: (A) an IVGTT, performed after 12 weeks of HFFD feeding. As described in the method, at the end of the protocol, rabbits were intravenously injected with a glucose bolus, then plasma (B) glucose and were evaluated. (B) The AUC_*Glu*_ of each curve from the IVGTT was calculated to compare the differences between the two groups. (C) Changes in plasma glucose levels 2 hours after the bolus were determined. (D) Insulin metabolism was assessed by calculating the Homeostasis model assessment of insulin resistance (HOMA-IR). Data were expressed as the mean ± SEM. ** *p* < 0.01 or * *p* < 0.05 HFFD vs. control group. $ $ *p* < 0.01 or $ *p* <0.05 12th week vs. baseline (n = 9 for control and n = 12 for HFFD).

Fasting insulinemia significantly increased in the HFFD group (12th week vs. Baseline, *p* < 0.03) and almost significantly increased when comparing the two groups together at the end of the protocol (HFFD vs. Control, *p* = 0.0504). The HOMA-IR significantly increased amongst individuals of the HFFD group (12th week vs. Baseline, *p* < 0.02) and when comparing the two groups together at the end of the protocol (HFFD vs. Control, *p* < 0.03) (Fig 1D). Moreover, fasting plasma glucose levels did not show any significant change (Table 1). However, the AUC_Glu_ from the IVGTT significantly increased when comparing the HFFD to the control group at the end of the protocol (*p* < 0.02) (Fig 1B). The Glycaemic levels obtained 2h after glucose injection also significantly increased in the HFFD group along time (12th week vs. Baseline, *p* < 0.05) and when comparing the HFFD to the control group at the end of the protocol (*p* < 0.02) (Fig 1C). In brief, both insulin sensitivity (increase in fasting insulinemia and HOMA-IR) and glucose tolerance (increased AUC_Glu_ from the IVGTT and the glycaemic levels 2-hours post-glucose injection) decreased in the HFFD-fed group.

### Cardiovascular function assessment

**Fig 2:**
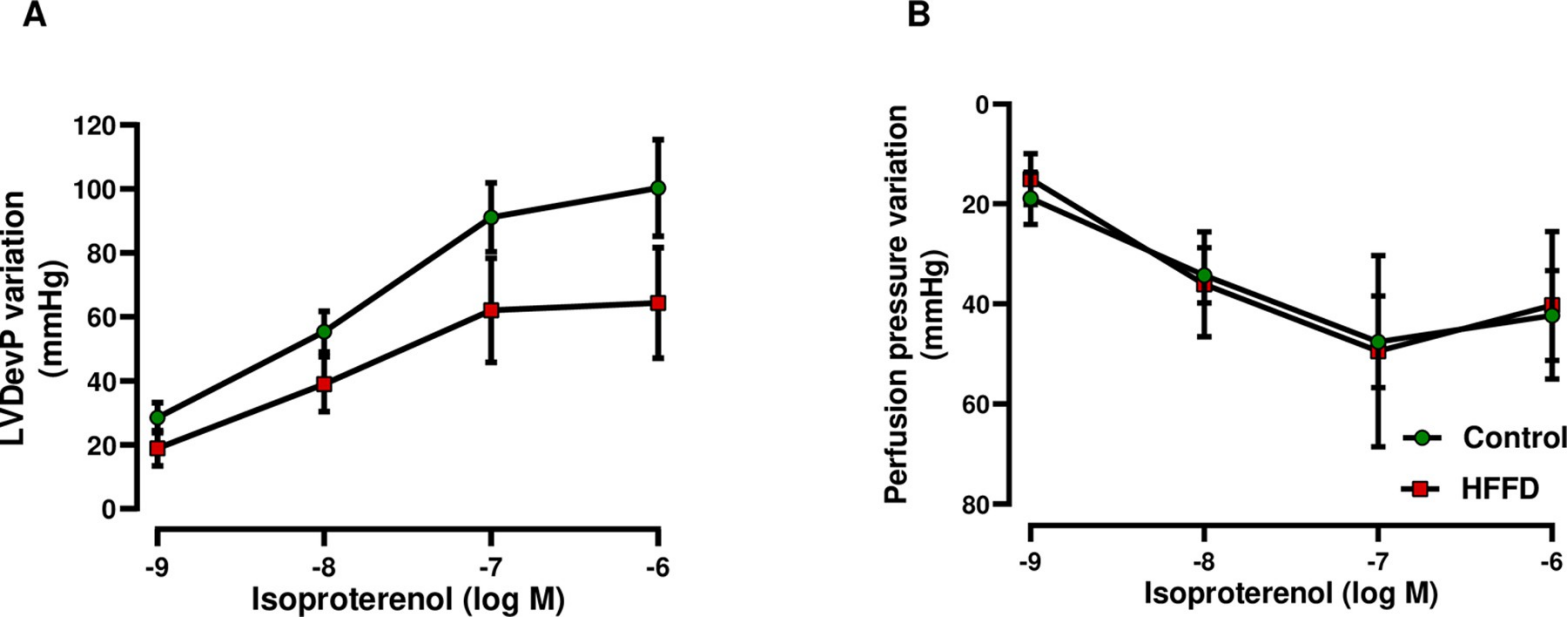
Effects of ß-adrenoceptor stimulation on cardiac function. Cardiac parameters were assessed after ß-adrenoceptor stimulation by building non-CCRCs to isoproterenol (10^−9^ to 10^−6^ M). The (A) LVDevP (left ventricular developed pressure) and (B) perfusion pressure, reflecting cardiac inotropy and coronary vasodilation, respectively, were evaluated. Data are represented as mean ± SEM and are analyzed using LME analysis (n = 6 for Control and n = 6 for HFFD).

To determine the effects of ß-adrenoceptor stimulation on cardiac inotropy and coronary vasodilation, non-CCRCs to isoproterenol were constructed (Fig 2). Our results showed that isoproterenol-induced positive inotropy exhibited a strong decreasing trend in HFFD-fed rabbits (*p* = 0.08) compared to the control group (Fig 2A). In terms of coronary vasodilation, we observed no difference between groups (Fig 2B).

**Fig 3:**
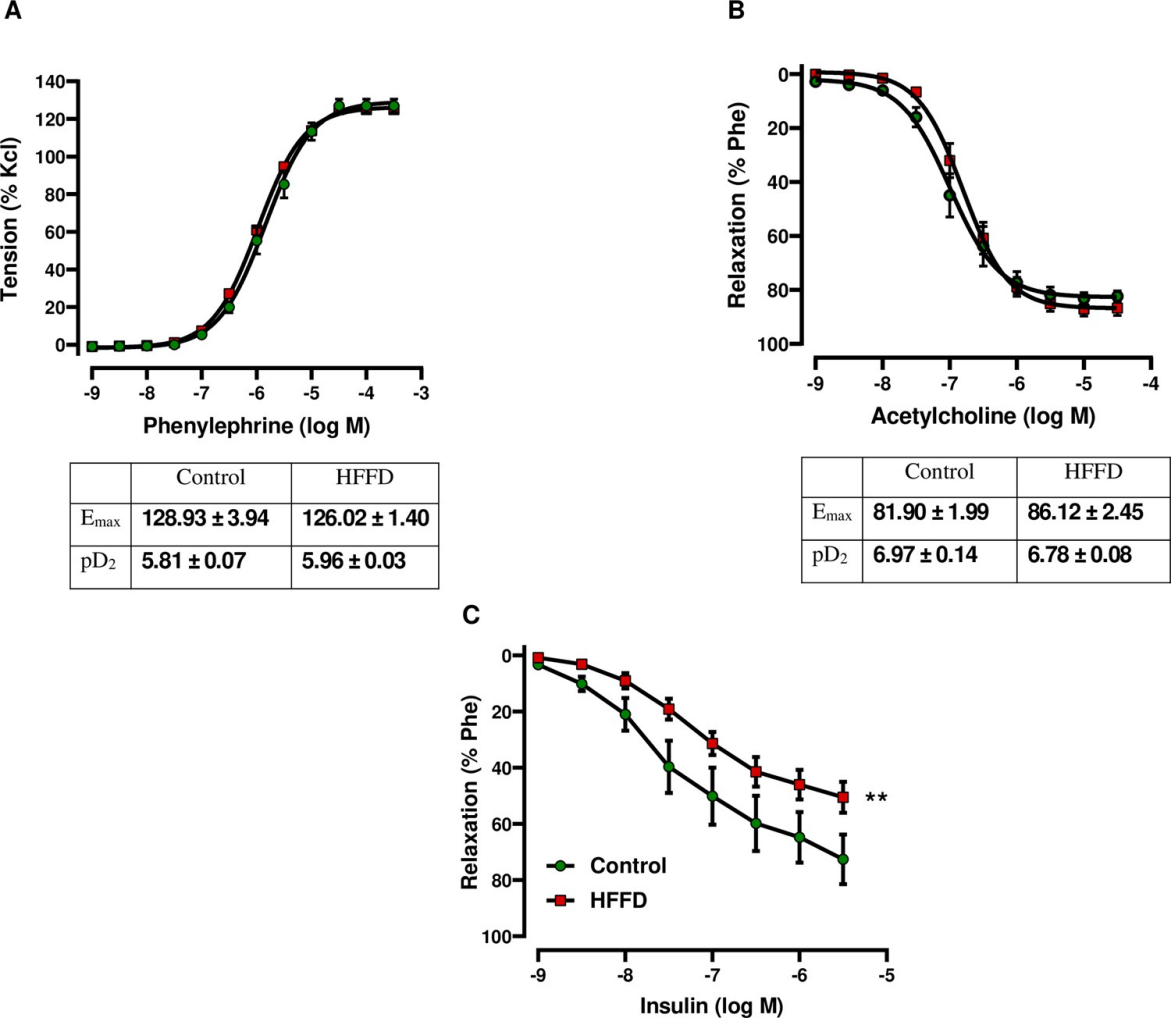
Effect of HFFD feeding on carotid vasoreactivity. CCRCs to (A) Phe, (B) Ach and (C) Ins were used to assess the vascular reactivity. (A) Carotid contractile response was evaluated using Phe (10^−10^ to 3.10^−5^). Meanwhile, relaxant responses were assessed by constructing CCRCs to: (B) Ach (10^−9^ to 3.10^−5^ M) and (C) Ins (10^−9^ to 3.10^−6^ M) on Phe-precontracted carotid rings. The calculated contraction and relaxation percentages are relative to the maximal changes from KCl (contraction) and Phe (precontraction), respectively. Emax and pD2 represent the maximal contractile response and the potency, respectively. Data are expressed as mean ± SEM and analyzed using NLME for Phe and Ach and LME for Ins. * *p* < 0.05, ** *p* < 0.01 HFFD vs. Control (n = 9 for Control and n = 12 for HFFD).

To assess the vascular reactivity (vasoconstriction and vasorelaxation), CCRCs were built (Fig 3). We observed no change in terms of Phe-induced contraction (Fig 3A). The endothelium-dependent Ins-induced vasorelaxation was significantly lower in the HFFD group (*p* < 0.01 vs. Control) (Fig 3C). On the other hand, we observed no difference neither in terms of E_max_ nor in terms of pD_2_ values, corresponding to endothelium-dependent Ach-induced relaxation (Fig 3B) and endothelium-independent relaxation response to sodium nitroprusside (SNP, 10^−10^ to 3.10^−5^ M) (S1 Fig).

### Aortic atheroma plaques evaluation

**Fig 4:**
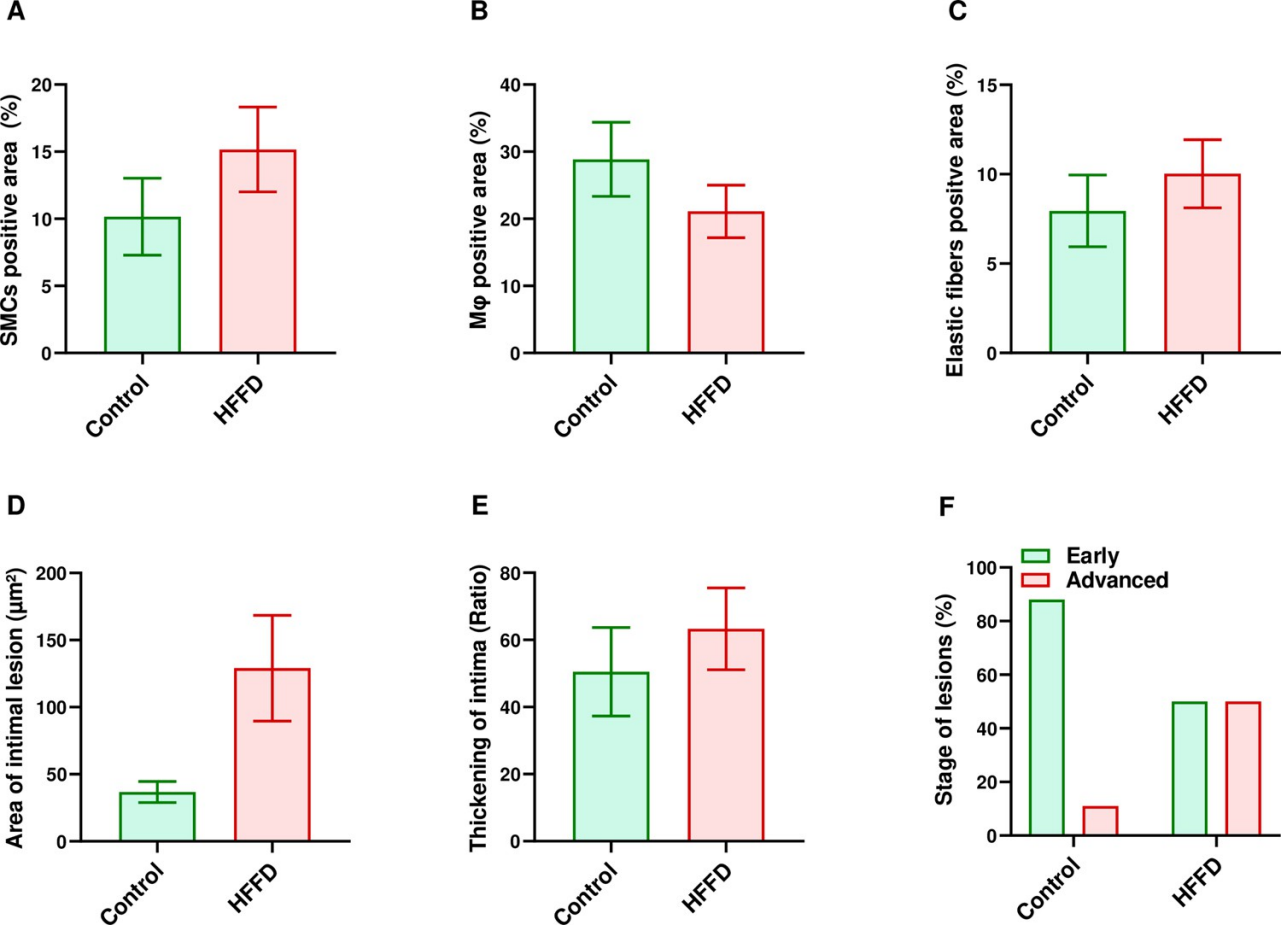
Quantification of aortic atherosclerosis following 12 weeks of HFFD feeding. Positively immuno-stained area of: (A) SMCs (detected using an anti-HHF35 antibody), (B) Mφ (detected using an anti-RAM11 antibody) and (C) elastic fibers (detected using the Orceine stain), were quantified using an image analysis system as described in the method. The same image analysis system was used to determine, (D) the intimal lesion area. Whereas, (E) the thickening of intima was determined by dividing the area of intimal lesion by the length of media. (F) Plaques were classified into early (type II and III plaques) and advanced (type IV and V plaques) stage lesions. Data in all graphs are represented as the mean ± SEM except for the stage of lesions, which was expressed in percentages. Statistical analysis was performed using one-way Anova for all parameters except for stage of lesions, for which a Chi square test was used (n = 9 for Control and n = 12 for HFFD).

We observed no change regarding the average thickening of the intima. The area of intimal lesion increased in the HFFD group compared to the control group; however, the increase was not significant. SMCs, Mφ and elastic fibers were not significantly different when comparing the HFFD to the control group. We observed a clear trend towards advanced lesions in the HFFD (*p* = 0.0614 vs. Control) when evaluating the stage of lesions (Fig 4).

We found an almost significant increase in the presence of extracellular lipid deposition and fibrous cap (*p* = 0.0567 and *p* = 0.0502, respectively) in the HFFD group (vs. Control). Regarding the extent of mineralization, lipid core severity and collagen deposition, there was no statistically significant difference between the two groups. Nevertheless, we did frequently observe marked and severe collagen deposition in the HFFD group and rarely in the control group. In terms of plaques types, most of plaques observed in the control group were either classified as type II (4/9) or as type III (3/9) with none type V plaques. In contrast, 50% of the plaques (6/12) were classified as type V plaques in the HFFD group. In the current study, only type II, III, IV and V lesions were observed (absence of type I and VI) (Table 2).

**Table 2:** Classification of atheroma plaques.

Histologic parameter evaluated	Classification	Control (n = 9)	HFFD (n = 12)	*p* value Control vs. HFFD
**Extracellular lipids**	**Absence**	3 (33.3%)	4 (33.3%)	0.0567
	**Grade 1**	5 (55.6%)	1 (8.3%)	
	**Grade 2**	1 (11.1%)	3 (25.0%)	
	**Grade 3**	0	4 (33.3%)	
	**Grade 4**	0	0	
**Fibrous cap**	**Absence**	7 (77.8%)	6 (50.0%)	0.0502
	**Grade 1**	2 (22.2%)	0	
	**Grade 2**	0	5 (41.7%)	
	**Grade 3**	0	1 (8.3%)	
	**Grade 4**	0	0	
**Mineralization**	**Absence**	8 (88.9%)	9 (75.0%)	0.43
	**Grade 1**	1 (11.1%)	3 (25.0%)	
	**Grade 2**	0	0	
	**Grade 3**	0	0	
	**Grade 4**	0	0	
**Lipid core**	**Absence**	6 (66.7%)	5 (41.7%)	0.2753
	**Grade 1**	2 (22.2%)	1 (8.3%)	
	**Grade 2**	1 (11.1%)	4 (33.3%)	
	**Grade 3**	0	2 (16.7%)	
	**Grade 4**	0	0	
**Collagen deposition**	**Absence**	1 (11.1%)	0	0.1888
	**Grade 1**	1 (11.1%)	0	
	**Grade 2**	4 (44.4%)	2 (16.7%)	
	**Grade 3**	2 (22.2%)	7 (58.3%)	
	**Grade 4**	1 (11.1%)	3 (25.0%)	
**Type of plaque**	**No plaque**	1 (11.1%)	0	0.09
	**Type II**	4 (44.4%)	4 (33.3%)	
	**Type III**	3 (33.3%)	2 (16.7%)	
	**Type IV**	1 (11.1%)	0	
	**Type V**	0	6 (50.0%)	

A semi-quantitative scale from 0 to 4 was used to grade the presence of extracellular lipid, fibrous cap, mineralization, lipid core in addition to collagen’s deposition (detected using green Masson’s trichrome stain). Types of plaques were classified according to the guidelines of the American Heart Association. The results were expressed as frequency and percentage. Statistical analysis was performed using a Chi-square test.

### Arterial stiffness measurement

**Fig 5:**
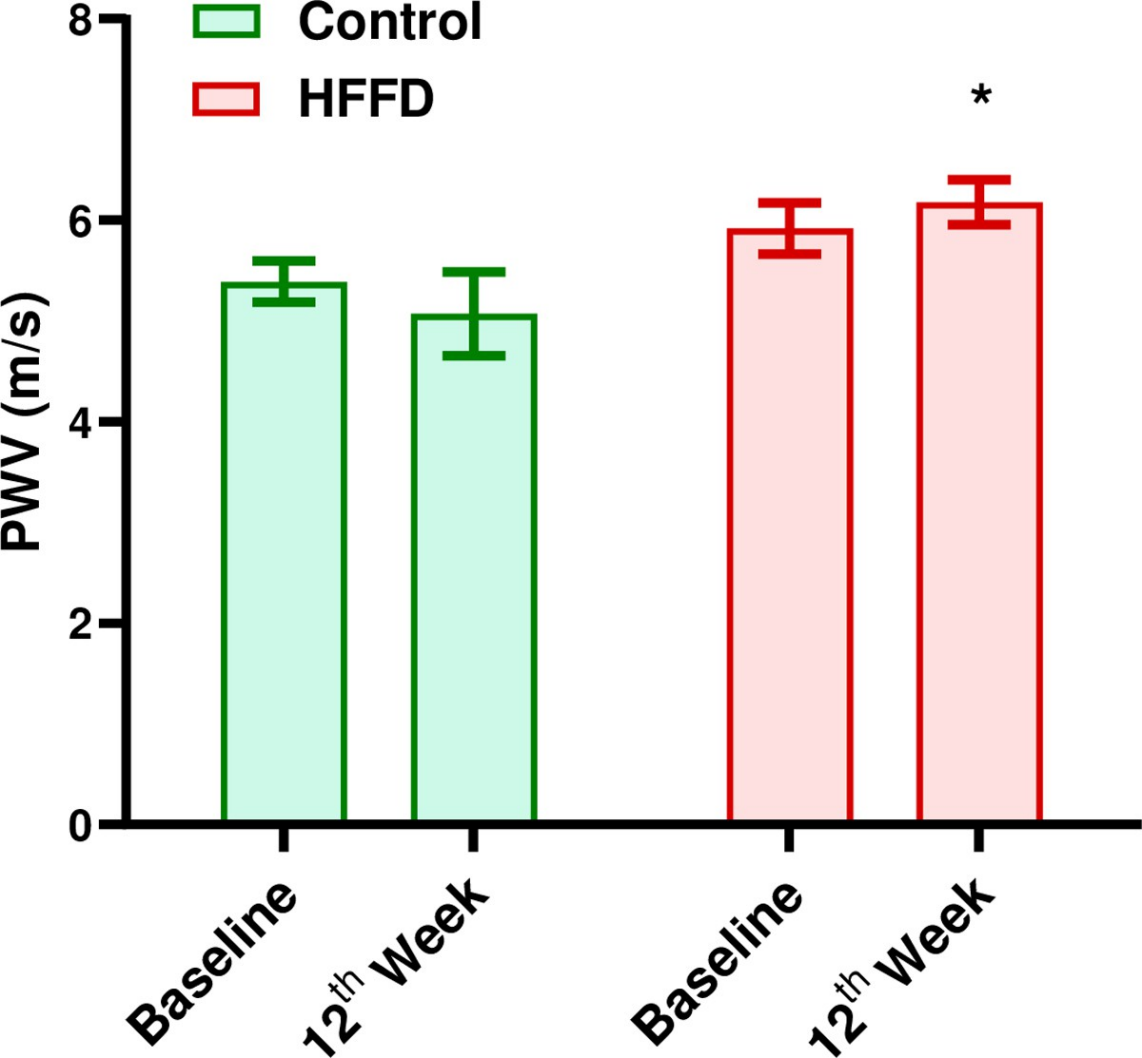
Effect of 12 weeks of HFFD feeding on arterial stiffness. The PWV was measured at the beginning and end of the protocol in order to assess the arterial vascular elasticity. It was calculated using the following formula: PWV (m/s) = Δx/Δt. Data are expressed as mean ± SEM. Repeated measures two-way Anova test was used for statistical analysis. n = 9 for control, n = 12 for HFFD. * *p <* 0.05 vs. Control.

PWV measurements were performed, to assess the aortic stiffness (Fig 5). Statistical analysis showed a significant difference between the control and HFFD group at the end of the protocol (HFFD vs. Control, *p <* 0.02). However, this difference cannot be taken into consideration given that the PWV almost did not change amongst individuals of the HFFD. This indicates that this difference is not related to the HFFD feeding per se but to a difference that already existed between individuals from the control and the HFFD groups at the beginning of the protocol.

### Gut microbiota analysis

**Fig 6:**
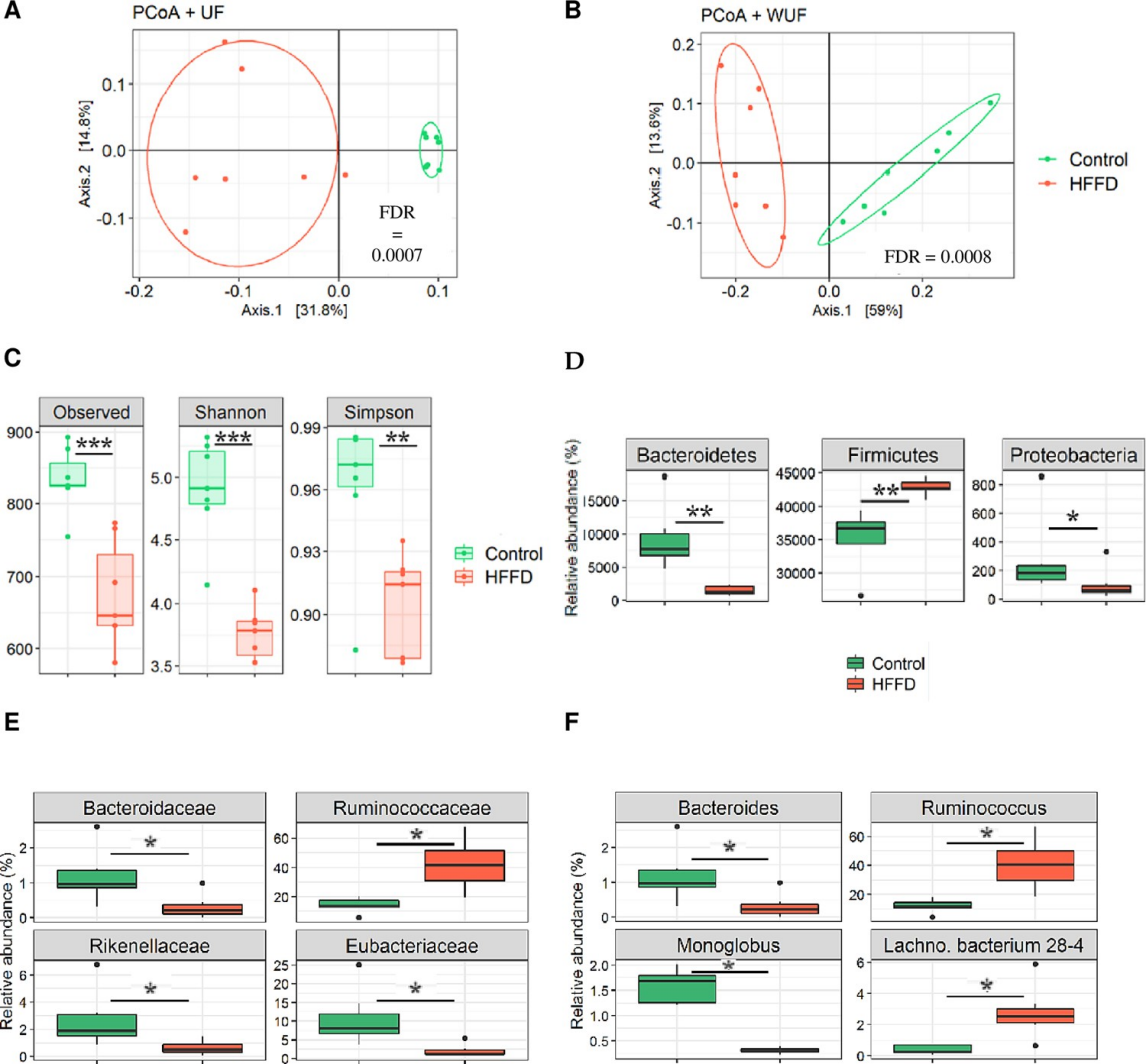
Gut bacterial community analysis by 16S rRNA gene high-throughput sequencing. Principle coordinate analysis (PCoA) based on A) Unifrac (UF) and B) weighted Unifrac (WUF) distance matrices, reveals useful information about the phylogenetic relationship and composition of bacterial microbiota in the different animal groups. C) Alpha diversity, which takes into account the community richness, evenness and diversity, was measured by three different indexes: Observed number of OTUs, Shannon and Simpson. Differences in relative abundance of bacterial taxa at the D) Phylum, E) Family and F) Genus levels. Data are represented as box plots. * *FDR* < 0.05, ** *FDR* < 0.01 and *** *FDR* < 0.001 (n = 7 for Control and n = 7 for HFFD).

PCoA based on the Unifrac (UF) and Weighted Unifrac (WUF) dissimilarity matrices represent distances between samples in function of their microbial composition (presence/absence) and relative abundances, respectively. Weighted UniFrac incorporates these abundances when calculating shared/unshared branch lengths to calculate distance, so the impact of low-abundance features is reduced. Unweighted UniFrac is more sensitive to differences in low-abundance features. So both are useful to interpret together. When comparing the UF and WUF distances between the two microbial communities of the control and HFFD groups, the differences were highly significant (*FDR* < 0.001 and *FDR* < 0.001, respectively). This means that samples from the same group of rabbits (control) clustered together and separately from samples belonging the other group of rabbits (HFFD) in the plot, indicating dissimilarities, in terms of both microbial composition and relative abundances, between communities belonging to the control and those belonging to the HFFD group (Fig 6A and 6B). As measured by the observed number of OTUs, Shannon and Simpson indexes, the intestinal microbial richness, evenness and diversity significantly decreased in the HFFD group compared to the control group (*FDR*< 0.001, *FDR* < 0.0001, *FDR* < 0.01, respectively) (Fig 6C). This indicates that the overall microbial alpha diversity decreased in response to 12 weeks of HFFD feeding. The Shannon index is strongly influenced by species richness and by rare species, while the Simpson index gives more weight to evenness and common species. The use of both diversity indices adds more information of the diversity in ecosystems, which is unique for each community or sample analyzed. When we evaluated the microbial composition at the phylum level, three main phyla were present, with *Firmicutes* as the most abundant followed by *Bacteroidetes* and *Actinobacteria* (S3 Fig). The abundance of *Firmicutes*, the dominant bacterial phylum, significantly increased (*FDR* < 0.01); whereas, the abundance of *Bacteroidetes* and that of *Proteobacteria* significantly decreased (*FDR* < 0.01 and *FDR* < 0.05, respectively) in the HFFD group compared to the control group (Fig 6D). We also evaluated the microbial composition at the Family level. We found a significant decrease in the abundance of *Rikenellaceae* (*FDR* < 0.05) and *Bacteroidaceae* (*FDR* < 0.05) on one hand; and a significant increase in *Ruminococcaceae* (*p* < 0.05), on the other hand (Fig 6E). At the genus level, *Ruminococcus* and *Lachno*. *Bacterium 28–4 (L*. *Bacterium 28–4)* significantly increased (*FDR* < 0.02 and *FDR* < 0.02) and *Bacteroides* significantly decreased (*FDR* < 0.03) in the HFFD group compared to the control group, respectively (Fig 6F).

**Fig 7:**
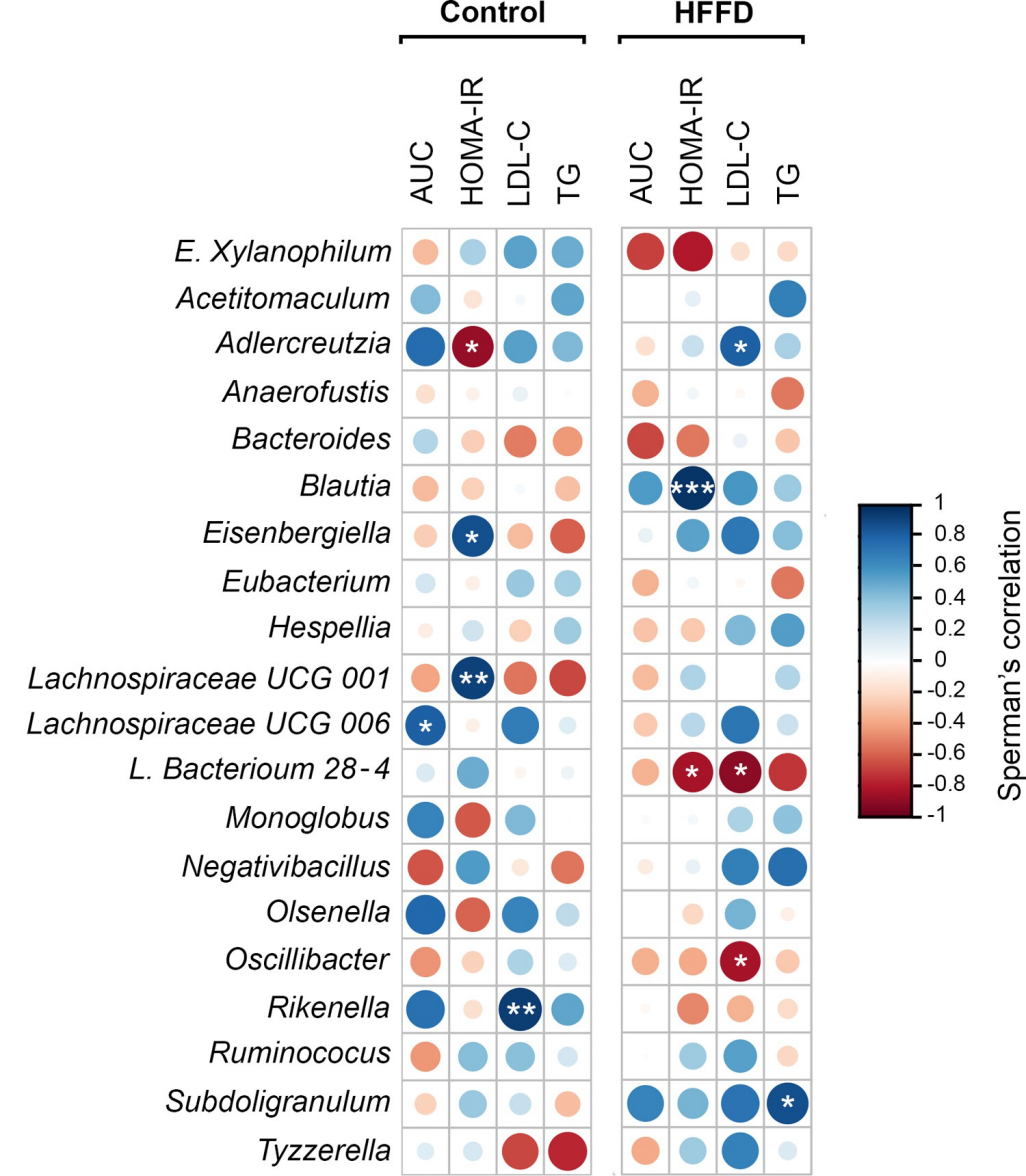
Correlation heatmap representing correlation coefficients between changes in bacterial abundance (at the genus level) and the host’s parameters (AUC_GTT_, HOMA-IR, LDL-C and TG). Associations were performed in R based on Spearman’s correlation. The corrplot and Hmisc package were used to produce the correlation matrix. Positive correlations are displayed in blue and negative correlations in red color. Color intensity and the size of the circle are proportional to the correlation coefficients. Correlation results were considered statistically significant at p value. * *FDR* < 0.05, ** *FDR*< 0.01 and *** *FDR* < 0.001 (n = 7 for Control and n = 7 for HFFD). AUC, HOMA-IR, LDL-C and TG denote area under the curve, homeostasis model assessment of insulin resistance, low density lipoprotein cholesterol and triglyceride, respectively.

We observed that rabbits belonging to the HFFD group represented a significant increase in the AUC_GTT_, HOMA-IR, LDL-C and TG levels as well as alterations in gut microbiota compared to the control group. Thus, we further analyzed whether there was a correlation between these abovementioned parameters and the shift in gut microbiota composition at the genus level (Fig 7). *Blautia* was significantly positively correlated with HOMA-IR (*FDR* < 0.001). *L*. *Bacterium 28–4* was significantly negatively correlated with both HOMA-IR and LDL-C (*FDR* < 0.05 and *FDR* < 0.02, respectively). *Oscillibacter* significantly negatively correlated with LDL-C (*FDR* < 0.05). *Subdoligranulum* significantly positively correlated with triglycerides (*FDR* < 0.03) (Fig 7).

## Discussion

In the current study, we explored the changes in metabolic and cardiovascular parameters, as well as in gut microbiota composition, related to a combination of two main factors of the MetS, dyslipidemia and IR. To produce this combination, we subjected the Watanabe rabbit, an animal model of dyslipidemia and atherosclerosis, to a HFFD, known for its ability to induce IR [26]. Our purpose was to induce IR rather than obesity; thus, the HFFD contained the same amount of calories (isocaloric) as the control diet (normal chow). The latter is protein- and fiber-rich, whereas the HFFD is rich in sugar and fat, but reduced in protein and fibers.

Consequently, we found no difference neither in terms of weight gain nor in terms of abdominal circumference, between control and HFFD individuals. Thus, increases in these two parameters were probably age-dependent and not specifically related to the HFFD feeding.

HFFD feeding significantly increased both the fasting plasma levels of insulin and HOMA-IR, two indicators of IR. These results along with the absence of increased weight and abdominal circumference are in consistence with several studies, that demonstrated that consumption of a normal number of calories, whether of a high fructose diet or of a high fructose-fat diet, induces IR without obesity in rabbit models [26, 46]. The HFFD-induced IR state is related to lipogenic effect of fructose. It has been demonstrated that a high fructose level stimulates visceral adipose deposition leading to an increased FA flux and lipid accumulation in insulin-sensitive tissues and thereby to impaired insulin signaling through the IRS1-PI3K-Akt (insulin receptor substrate 1-phosphoinositide 3-kinase-protein kinase B) pathway [10, 47]. Then, the impaired insulin signaling results in decreased translocation of GLUT 4 to the cell surface and thereby to reduced glucose uptake, in insulin-sensitive tissues [48, 49]. This explains our results regarding the decrease in glucose tolerance in the HFFD-fed group. When challenged with a glucose bolus (Fig 1), HFFD-fed rabbits exhibited delayed capacity for clearing glucose from the circulation.

It has been shown that elevated fructose concentrations enhances the de novo lipogenesis rate [48]. A high fructose flux, to the liver, increases TG synthesis and/or decreases TG clearance ultimately leading to increased production of very low density lipoprotein (VLDL) particles [50, 51]. Moreover, high fat-feeding increases the availability of exogenous lipids, stimulating lipogenesis [52]. This explains our results regarding the increased TG levels found in the HFFD group [53]. The excess of VLDL secretion then delivers higher levels of FAs and TG to skeletal muscle and adipose tissue, further inducing IR [54].

It has been described that, in states of obesity, fasting leads to mobilization of energy stores (TG) in adipose tissue via lipolysis, resulting in increased FFA levels [55]. Therefore, the unchanged FFA levels might be explained by the absence of both, excessive weight gain and increased abdominal circumference in the HFFD-fed subjects.

Even though control Watanabe rabbits spontaneously develop hypercholesterolemia due a LDLr mutation [22, 25]; we still found that 12 weeks of HFFD feeding leads to an extensive hypercholesterolemia. This can be explained by an increase in TG-enriched VLDL production (as described earlier) due to the long-term HFFD-feeding. These VLDL particles would then undergo lipolysis yielding higher levels of LDL-C particles and subsequently higher plasma cholesterol levels in the circulation. It is highly probable that in Watanabe rabbits, the reduced clearance of lipoproteins (mutated LDLr) underlies the further increase in blood cholesterol levels.

Besides its well established relation with dyslipidemia, atherosclerosis has also been reported to have a strong connection with IR [56, 57]. Hence, we evaluated the evolution of aortic atherosclerotic plaques in terms of quality and quantity. We observed a qualitative amplification of atherosclerosis in individuals with combined dyslipidemia-IR that is likely due to increase hypercholesterolemia in the HFFD group. Moreover, IR, per se, has been found to increase plasma lipid levels [58]. Under physiological conditions, insulin decreases VLDL-TG and apo-lipoprotein B (apoB) production and enhances apoB degradation [59]. During IR states, insulin’s physiological effects are counteracted leading to increased production and secretion of lipids [60]. Hence, it is plausible that IR together with elevated plasma lipids play combinational roles in the worsening of atherosclerosis by making the lesions become more vulnerable/susceptible to complications, in HFFD-fed WHHL rabbits [26]. We also measured the pulse wave velocity in order to assess the arterial stiffness which has been proven to be correlated with the MetS in humans and experimental animal models [61]. In contrast to others who found that PWV increases in an animal model of diet-induced metabolic syndrome [62], our results showed no significant difference between the two groups (Fig 5). This might be related to the fact that our protocol only lasted for 12 weeks. It is highly possible that the evolution of increased arterial stiffness is at an early stage and needs longer than 12 weeks before it becomes detectable through PWV measurements.

It has been proposed that the hyperinsulinemia (during IR) and dyslipidemia lead to sympathetic nervous system (SNS) overdrive [63, 64]; which thereby leads to decreased myocardial contractile response to β-stimulation [65]. We evaluated the effects of β-adrenergic stimulation on cardiac inotropy and coronary vasodilation. As previously stated, IR might lead to cardiac dysfunction and remodeling [66] and as proven, cardiac IR reduces the metabolic efficiency of the heart, leading to contractile dysfunction in mice [67]. Our results showed a strong decreasing trend in the positive inotropy in HFFD group, indicating a likelihood implication of IR and/or dyslipidemia. Alterations in the β-adrenergic system may occur either at the receptor or post-receptor intracellular signaling level, i.e. calcium (Ca^2^⁺) handling proteins [68–71].

A strong link between IR and endothelial dysfunction (ED) has been well established [49]. Thus, we evaluated the reactivity of the carotid artery. The HFFD did not affect relaxation to Ach, in contrast to other studies who found that high fructose and/or high fat feeding leads to impaired Ach-induced vasorelaxation [72]. It has been found that westernized diet-induced oxidative stress results in reduced NO production and/or increased NO sequestration and inactivation thereby leading to ED [72]. A possible hypothesis behind the intact Ach-induced relaxation is that adaptive mechanisms could have developed to compensate/offset decreased NO-dependent relaxations following reduced availability of NO and/or decreased eNOS activity [73–75]. Meanwhile, our results showed a diminished insulin-mediated vasorelaxation (mainly endothelium-dependent) [76], in the HFFD-fed group, probably related to the diet-induced IR-state. In physiologic states, insulin stimulates NO release from the vascular endothelium through PI3K‐Akt‐mediated phosphorylation of eNOS, leading to vasorelaxation. During IR, signaling through this pathway is downregulated leading to a compromised vasorelaxant effect of insulin [77]. It is possible that the duration of exposure to the HFFD was not severe/long enough to induce an impaired response to Ach and conversely enough to functionally impair the responsiveness to insulin [78]. Moreover, the vasodilatory actions of insulin are well known for their contribution to insulin delivery and glucose-uptake in insulin-sensitive tissues [79]. Thus, vascular IR contributes to an impaired insulin‐stimulated glucose uptake and thereby to decreased glucose tolerance.

Evidence from human and animal studies support a link between the gut microbiome and several components of the MetS [15, 80]. Moreover, it is believed that changes in diet and activity patterns alter the gut microbiome composition/diversity, leading to changes in the metabolic profile of microbiota and thereby to the onset of disease e.g. MetS [8, 81]. Thus, we explored the gut microbial changes that occurred following long term HFFD-feeding to Watanabe rabbits.

Our results showed that 12 weeks of HFFD-feeding led to a shift in overall intestinal microbial composition, characterized by changes in abundance of dominant bacterial Phyla, and diversity (richness and evenness); indicating a strong impact of dietary fat and carbohydrates on the gut microbiota. In consistence with our results, it has been demonstrated that low richness in gut microbiota, reflecting a reduced microbial diversity correlates with several components of the MetS, namely, IR and dyslipidemia [82].

In the current study, HFFD-fed rabbits exhibited an increased *Firmicutes* abundance and a decreased *Bacteroidetes* abundance. Similar changes in microbiota composition have been observed in mice fed a “Western” diet [83]. Decreased Bacteroidetes abundance was reported, in germfree mice colonized with human microbiota, upon a dietary shift to a “Western” diet [84]. The *Firmicutes* to *Bacteroidetes* ratio, used as a relevant marker/hallmark of metabolic dysfunction [81, 85], increased from R = 3.39 in the control group to R = 28.24 in the HFFD group (S1A & S1B Table). This is in agreement with a recent study that found an increase in the *Firmicutes*/*Bacteroidetes* ratio in high-fructose/high-fat diet-fed hamsters [85]. *Firmicutes* were proposed as more effective in extracting energy from food than *Bacteroidetes* [86] which could have contributed to the IR-state in the HFFD-fed group. Even though no apparent/overall obesity was observed in individuals from the HFFD group, these rabbits still developed a state of IR indicating the presence of visceral obesity.

Moreover, *Bacteroidetes* were suggested to have a protective/balancing role against metabolic impairment in rodents and human studies; and more specifically the *Rikenellaceae* Family, was found to point towards more healthy metabolic states [87]. furthermore, in another study decreased *Rikenellaceae* abundance was found to be associated with a negative metabolic outcome e.g. increased BMI [88]. In accordance with those studies, we found a decreased abundance of the *Rikenellaceae* Family in rabbits belonging to the HFFD group, which is most likely related to the altered metabolic state in these rabbits. Our results also showed that HFFD-feeding leads to decreased abundance in another Family belonging to *Bacteroidetes* Phylum, *Bacteroidaceae* which, according to Lippert et al., inversely correlates with visceral obesity [87]. As mentioned earlier, in the present study, rabbits from the HFFD group likely exhibited excessive visceral (rather than subcutaneous) fat accumulation, associated with the IR state that occurred following 12 weeks of HFFD-feeding. On the other hand, Zeng et al. reported a negative correlation between *bacteroides* genus and serum lipids (TC, LDL-C and TG levels) [89]. Accordingly, the decrease in the *bacteroides* genus, found in our study, might be explained by the significant increase in the aforementioned lipid levels in the HFFD group. Conversely, we found neither positive nor negative correlations, when we further analyzed associations between decreased *Bacteroides* abundance and the increase in both LDL-C and TG levels in the HFFD group. It has been found that *Ruminococcaceae* were significantly higher in subjects with impaired glucose tolerance and that *Ruminococcus* genus, in particular, correlates with dysglycemia [90]. Hence, we hypothesized that both the increased *Ruminococcaceae* and *Ruminococcus* abundances in individuals from the HFFD group, in the current study, are likely related to the glucose intolerance/IR state. Unexpectedly, our results revealed no positive or negative correlations between increased *Ruminococcus* abundance and the increase in both AUC_GTT_ and HOMA-IR in the HFFD group_._

In contrast to our findings, consumption of diets rich in fat and sugars was found to increase the number of *Proteobacteria* Phylum in mice [91, 92]. Nonetheless, others stated that unfavorable lipid profiles/dyslipidemia, correlates with low microbial diversity and lower abundance of many taxa from phyla *Proteobacteria* and *Bacteroidetes*. Thus, the decreased microbial diversity and abundance of both these phyla [93], in our study, is most probably related to the worsened dyslipidaemic-state in response to the 12-weeks HFFD feeding.

*L*. *Bacterium 28–4*, a member of the *Lachnospiraceae* family was negatively correlated with both HOMA-IR and LDL-C in our study. Data on the *L*.*Bacterium 28–4* genera are scarce however several studies positively correlated high abundances of Lachnospiraceae (*Firmicutes* Phylum) with metabolic disturbances (glucose and/or lipid metabolism) [87, 94–96]. Moreover, our results revealed an association between *Blautia* genus, another bacterium of the *Lachnospiraceae* family, and HOMA-IR, as previously found [97]. Indeed, increased abundance of *Blautia* correlated with pathological manifestations such as glucose intolerance in humans [98] and IR in Zucker diabetic fatty (ZDF) rats [99] and was linked to metabolites, indicating an unfavorable metabolic state [100]. This might be related to *Blautia*’s association with increased gut permeability [101]. In contradiction to our results, *Subdoligranulum* has been previously found to negatively correlate with an unhealthy metabolic profile e.g. increased fat mass [102]. Nevertheless, the negative correlation between cholesterol levels and *Oscillibacter*, found in our study, has been previously reported [103]. Furthermore, *Oscillibacter* has been found to be increased in response to reduced carbohydrate weight loss diet [104].

### Study limitations and future research direction

One limitation of the current study is the absence of result analysis based on sex, because of possible sex-dependent influence on the results in response to HFFD feeding. Therefore, results must be interpreted with caution. For ethical reasons, authors chose to work on small groups of rabbits and thus did not split animals based on gender. Another limitation is the lack of further vascular disease measurements e.g. inflammatory markers such as ICAM-1, VCAM-1, IL-8, MCP-1, etc. Furthermore exploring liver disease formation i.e. NASH or NAFLD would have been interesting in our animal model due to the existing association between the components of MetS (i.e. visceral obesity, insulin resistance and dyslipidemia) and NALD/NASH. In the future, the animal model used in our study can be further developed to explore new therapeutic strategies in the management of the MetS and its associated cardiovascular disorders.

## Conclusion

We demonstrated that long-term HFFD-feeding leads to the development of MetS by inducing an IR state and exacerbating dyslipidemia, and to a shift in overall intestinal microbial composition in WHHL rabbits. We also showed that, independently from obesity, combined IR-dyslipidemia underlies atherogenic (worsened aortic atherosclerosis) and deleterious cardiovascular effects (decreased cardiac contractility and reduced insulin-mediated vasorelaxation) and induces gut microbiota dysbiosis. The current study confirms that (isocaloric) HFFD-fed WHHL rabbit represents a promising diet based and time efficient experimental model of MetS, without obesity as a main factor.

## Supporting information

S1 Fig Cumulative concentration response curves to SNP (10^−10^ to 3.10^−5^ M) on Phe-precontracted carotid rings. n = 9 for control and n = 12 for HFFD.
(TIF)

S2 Fig Evaluation of aortic arch atheroma plaques development in response to 12 weeks exposure to HFFD. Pictures (scale bar = 100μm) of stained sections: (A) smooth muscle cells (green arrow: SMCs stained in brown), (B) elastic fibers (blue arrow: elastic fibers stained in red brown), (C) macrophages (black arrow: Mφ stained in brown) and (D) Fibrosis (orange arrow: collagen stained in green), are represented above. Statistical analysis was performed using one-way ANOVA.
(TIF)

S3 Fig Bacterial composition at the phylum level (16S rRNA gene high-throughput sequencing). (TIF)

S1 Table A & B. *Firmicutes* to *Bacteroidetes* (*Firmicutes*/*Bacteroidetes)* ratio in control and HFFD groups, respectively. (TIF)

S2 Table Composition of the standard chow and the high-fructose high-fat diets. (TIF)
