# Supplementary figures and images for: Long-term high-fructose high-fat diet feeding elicits insulin resistance, exacerbates dyslipidemia and induces gut microbiota dysbiosis in WHHL rabbits
Source: PLoS One. 2022 Feb 23;17(2):e0264215. doi: 10.1371/journal.pone.0264215 (PMC8865649; doi:10.1371/journal.pone.0264215)

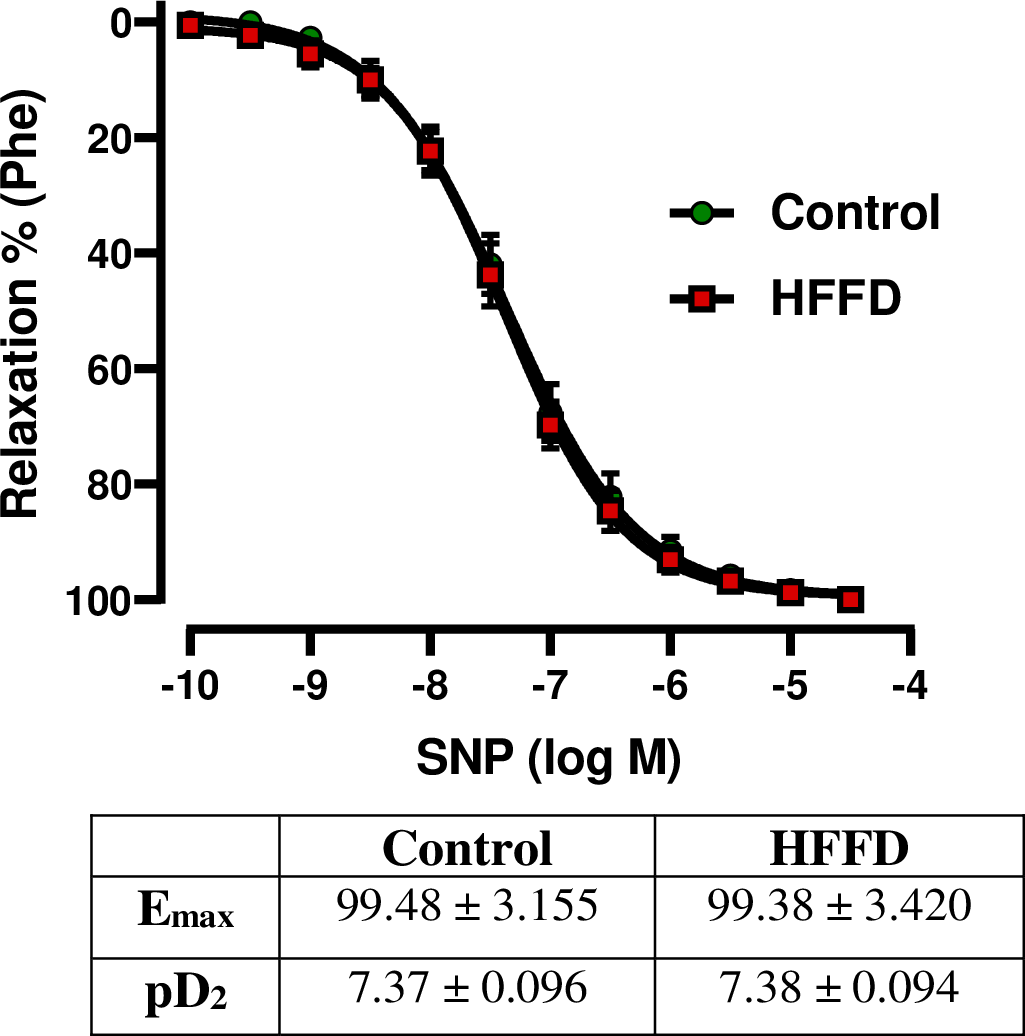

Supplement: S1 Fig — n = 9 for control and n = 12 for HFFD. (TIF) [file pone.0264215.s001.tif]

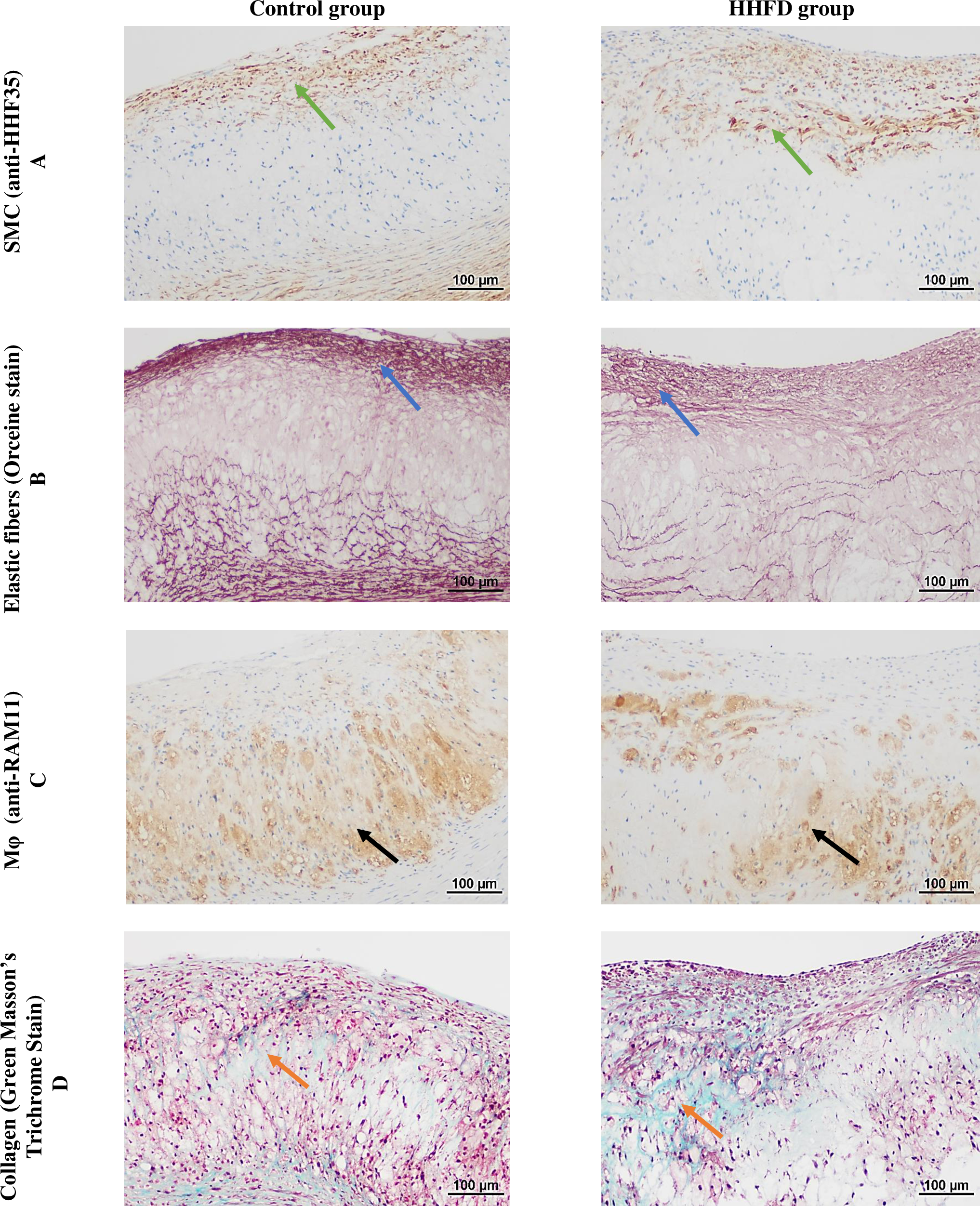

Supplement: S2 Fig — Pictures (scale bar = 100μm) of stained sections: (A) smooth muscle cells (green arrow: SMCs stained in brown), (B) elastic fibers (blue arrow: elastic fibers stained in red brown), (C) macrophages (black arrow: Mφ stained in brown) and (D) Fibrosis (orange arrow: collagen stained in green), are represented above. Statistical analysis was performed using one-way ANOVA. (TIF) [file pone.0264215.s002.tif]

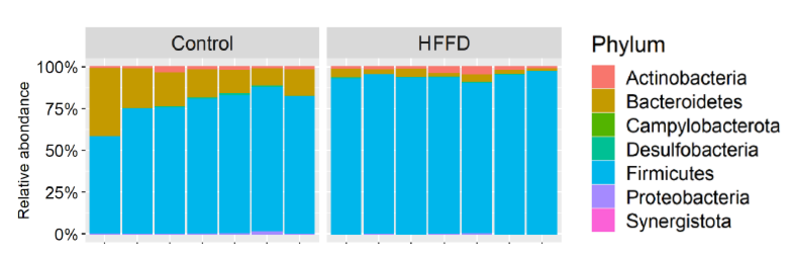

Supplement: S3 Fig — (TIF) [file pone.0264215.s003.tif]

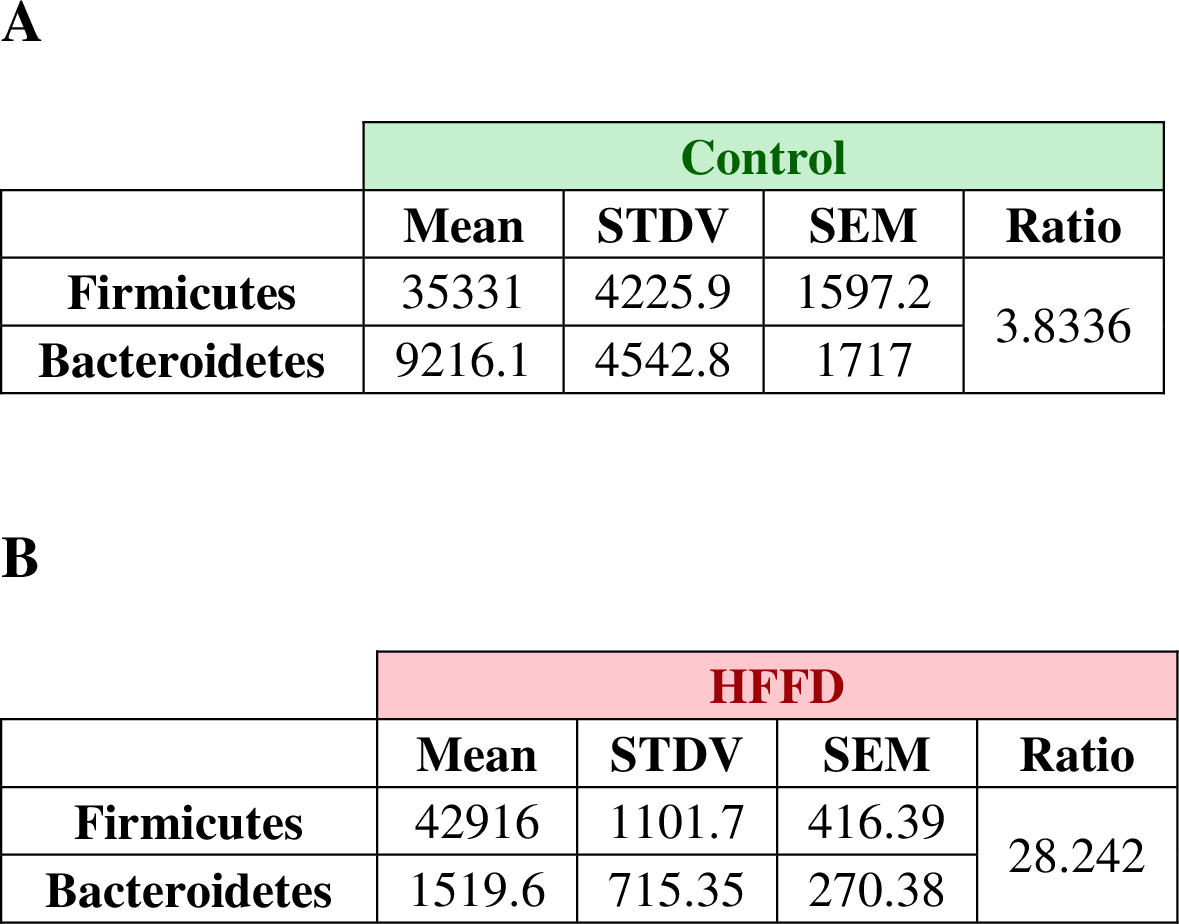

Supplement: S1 Table — (TIF) [file pone.0264215.s004.tif]

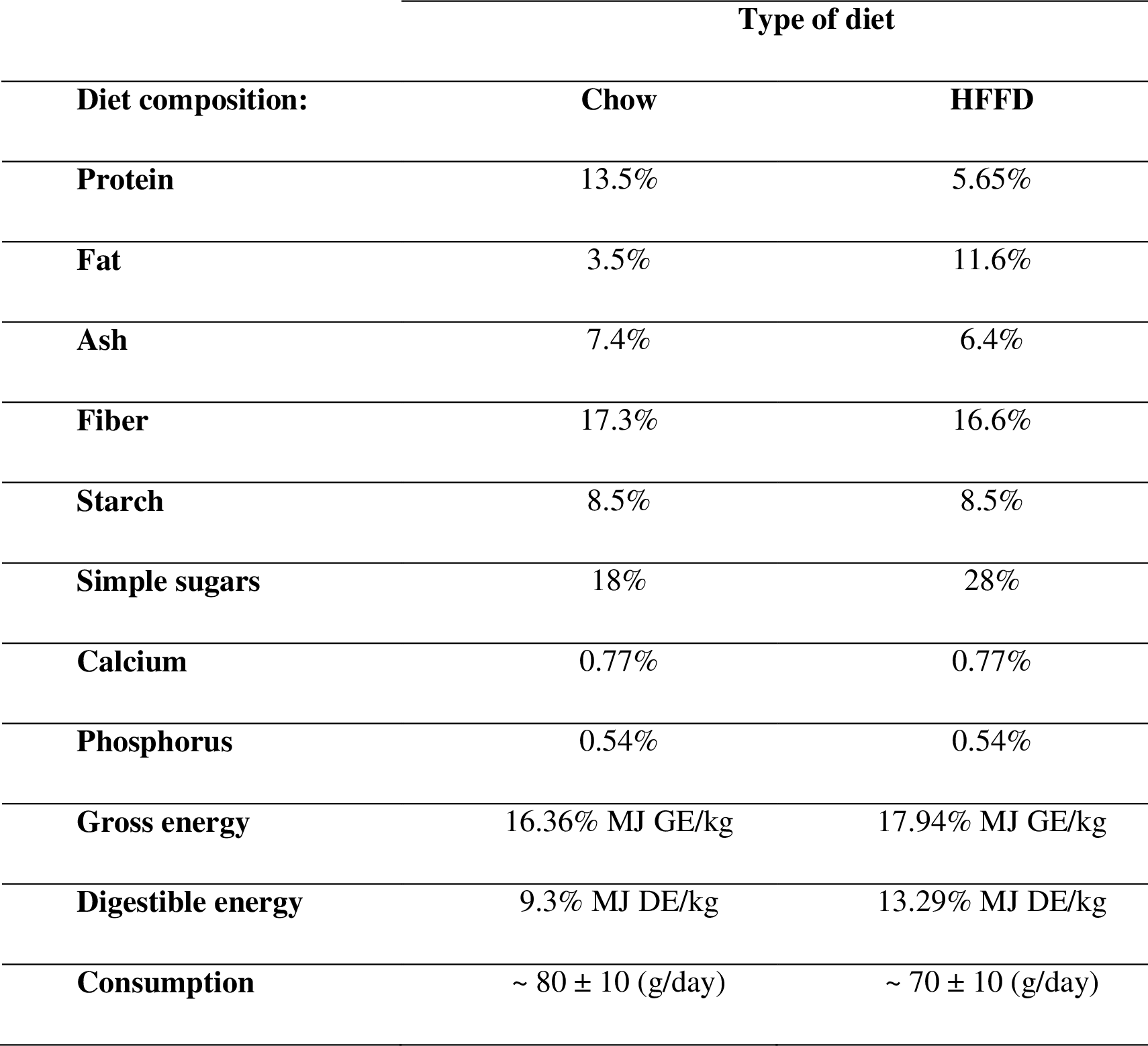

Supplement: S2 Table — (TIF) [file pone.0264215.s005.tif]
